# The Bidirectional Relationship between Tuberculosis and Diabetes

**DOI:** 10.1155/2017/1702578

**Published:** 2017-11-12

**Authors:** Ernest Yorke, Yacoba Atiase, Josephine Akpalu, Osei Sarfo-Kantanka, Vincent Boima, Ida Dzifa Dey

**Affiliations:** ^1^Endocrine & Diabetes Unit, Department of Medicine and Therapeutics, School of Medicine and Dentistry, College of Health Sciences, University of Ghana, Legon, Accra, Ghana; ^2^Directorate of Medicine, Endocrine and Diabetes Unit, Komfo Anokye Teaching Hospital, Kumasi, Ghana; ^3^Department of Medicine and Therapeutics, School of Medicine and Dentistry, College of Health Sciences, University of Ghana, Legon, Accra, Ghana

## Abstract

The burden of tuberculosis (TB) especially in developing countries continues to remain high despite efforts to improve preventive strategies. Known traditional risk factors for TB include poverty, malnutrition, overcrowding, and HIV/AIDS; however, diabetes, which causes immunosuppression, is increasingly being recognized as an independent risk factor for tuberculosis, and the two often coexist and impact each other. Diabetes may also lead to severe disease, reactivation of dormant tuberculosis foci, and poor treatment outcomes. Tuberculosis as a disease entity on the other hand and some commonly used antituberculous medications separately may cause impaired glucose tolerance. This review seeks to highlight the impact of comorbid TB and diabetes on each other. It is our hope that this review will increase the awareness of clinicians and managers of TB and diabetes programs on the effect of the interaction between these two disease entities and how to better screen and manage patients.

## 1. Introduction

TB infections continue to be a concern worldwide and it remains a deadly communicable disease. The World Health Organization (WHO) estimated that 10.4 million new cases of TB occurred and 1.4 million died from the disease in 2015 despite several preventive strategies to reduce the burden and impact [1]. Over 70% of these new cases occurred in developing countries, with the African region experiencing the highest rate of death relative to population [1]. The International Diabetes Federation (IDF) estimates a current worldwide diabetes disease burden of about 415 million, which is projected to hit 642 million by 2040 (over 60% increase) [2]. Developing countries are expected to experience the most increases driven mainly by type 2 diabetes [2]. This expected trend in the two conditions would also increase the interaction between them [1, 2].

Aside from the traditional risk factors which include poverty, malnutrition, overcrowding, and immunosuppression including HIV/AIDS, diabetes is increasingly being recognized as an independent risk factor for tuberculosis, and the two often coexist [3, 4]. Several studies around the world suggest that 5–30% of TB patients have concomitant diabetes mellitus [5].

This TB and diabetes association was recognized by Avicenia in as early as 1000 AD, when he noted that tuberculosis (called phthisis in Greek) was often associated with diabetes [6]. Also, an Indian saint, Yugimahamuni, described a cluster of symptoms for the TB/diabetes association (which he called meganoikal). These symptoms include obesity, glycosuria, thirst, incontinence, respiratory symptoms, and unconsciousness [7]. Subsequently, this interaction between TB and diabetes is increasingly being recognized and managed [3]. Diabetes and TB as separate disease entities impact negatively each other [8]. Whilst diabetes may lead to a more severe form of TB and affect its presentation, TB may lead to impaired glucose tolerance and hamper glycemic control [9].

## 2. The Interplay between Diabetes and Tuberculosis

### 2.1. Diabetes as a Risk Factor for TB

Diabetes is a risk factor for lower respiratory infections including TB. Despite the fact that TB is more associated with other immunosuppressive states like HIV infection, because of the greater numbers, diabetes remains a more significant factor for TB infections at the population level [3]. A review by Stevenson et al. reported that diabetes increases TB risk 1.5 to 7.8 times [10], whilst another meta-analysis by Jeon and Murray found that the relative risk for TB among diabetes patients was 3.11 [11]. In the latter review, among TB patients, diabetes prevalence ranged from 1.9% to as high as 35% after screening; and the highest rates were among regions of the world with the highest diabetes prevalence [11]. Again, an American study reported that the odds ratio of multidrug-resistant (MDR) TB associated with diabetes patients is 2.1 [12].

Although type 2 diabetes is more prevalent worldwide, the risk of tuberculosis in type 1 diabetes is three to five times [13, 14] higher due to relatively poorer control, lower body weight, and young age of affected persons [14].

Whilst it is not clear whether diabetes affects the presentation of TB, among diabetes patients, they tend to show more lower lobe involvement than their nondiabetic counterparts due to reactivation of old foci [8]. Again, some studies have reported lower rates of cavitation [12] whilst others reported higher rates [15–17]. TB associated with diabetes may also show higher rates of hemoptysis, fever, and atypical presentations compared with nondiabetics with TB [10, 12, 18].

Alisjahbana et al. reported in 2007 that TB patients who had diabetes had more symptoms, but with no evidence of more severe disease, and a higher percentage positive sputum for acid-fast bacilli after microscopic examination at two months compared with their nondiabetic counterparts (18.1% versus 10.0%). However, this was no longer statistically significant after adjustment for age, sex, BMI, study site, chest radiograph abnormalities, and sputum mycobacterial load before initiation of treatment. The study also reported that, after 6 months, 22.2% of the sputum of the diabetes patients grew* Mycobacterium tuberculosis *compared with 6.9% among controls (adjusted odds ratio: 7.65; *P* = 0.004) [12]. In a retrospective study involving TB patients from South Texas (USA) and northeastern Mexico they found out that patients with diabetes (identified by self-reporting) were more likely to remain positive at the first month (Texas cohort) or second month (Mexico cohort) of treatment [15]. Other studies have also shown a trend towards increased time to sputum conversion [19–22], whilst other have shown no relationship between diabetes and sputum conversion rate at the end of month two [21–24].

Diabetes increases the risk of failure and death combined, death, and relapse among patients with TB. A systematic review by Baker et al. reported that patients with diabetes have a risk ratio (RR) for the combined outcome of failure and death of 1.69 (95% CI: 1.36 to 2.12), whilst the RR of death during tuberculosis treatment among 23 unadjusted studies was 1.89 (95% CI: 1.52 to 2.36) [25]. Diabetes was also associated with an increased risk of relapse (RR: 3.89; 95% CI: 2.43 to 6.23) [25]. This review however did not find evidence for an increased risk of tuberculosis recurrence with drug-resistant strains among people with diabetes [25]. In a Brazilian study that was aimed at assessing the sociodemographic and clinical factors that may influence different outcomes of TB in patients with diabetes identified in the Brazilian national database from 2001 to 2011, it was found out that the development of MDR TB was more related to relapse (OR = 9.60, 95% CI: 6.07–15.14), previous default (OR = 17.13, 95% CI: 9.58–30.63), and transfer of treatment centers (OR = 7.87, 95% CI: 4.74–13.07) [26].

Diabetes can affect the pharmacokinetics of anti-TB drugs, especially rifampicin, by reducing their plasma concentrations [4]. However, there are conflicting reports on whether this affects the efficacy of TB treatment [4, 8]. Consequently, the regimen for treatment of TB among both diabetics and nondiabetics remains the same at the moment [3, 4].

### 2.2. TB as a Risk Factor for Diabetes

Whilst the bidirectional relationship between diabetes and TB has long been recognized, dedicated studies to assess whether TB increases the risk of diabetes are few [27–29]. TB can lead to impaired glucose tolerance (IGT) [29, 30] and new onset diabetes [9, 18, 29]. Generally, IGT normalizes after the TB has been successfully treated, but it remains a significant risk factor for developing type 2 diabetes in the future [31].

It is often difficult to conclusively label TB as the risk factor for newly discovered hyperglycemia or diabetes among previously unscreened TB patients [28, 29, 32]. Basoglu et al. [29], in a study of active TB patients without any history of diabetes mellitus, found glucose intolerance among 10.4% and diabetes in 8.6% in his cohort; and compared with a matched control group of community-acquired pneumonia, 17.4% were found to have diabetes and none of them had glucose intolerance. There was no significant difference between the two groups (*P* > 0.05). Oral glucose tolerance test (OGTT) results returned to normal in both TB and pneumonia groups after treatment. In a Nigerian study, a sequential follow-up of oral glucose tolerance tests on 54 patients with active TB patients, 42.6% were found to have abnormal results, of whom 5.6% had diabetes and 37.0% had IGT [28]. Three months after the antituberculosis medication, only one of the eight patients with impaired glucose tolerance at the second oral glucose tolerance test remained intolerant of glucose whilst only one patient was frankly diabetic. Again, among a predominantly white English population, a retrospective cohort analysis using data from two Oxford Record Linkage Study (ORLS) datasets from 1963 to 2005 was carried out by Young et al. [27]. They found out that whilst diabetes was associated with a two- to threefold increased risk of TB, there was no evidence that TB increases the risk of DM.

This infection-related hyperglycemia and some commonly used antituberculosis drugs such as rifampicin and isoniazid may lead to overdiagnosis of diabetes in previously unscreened TB patients [3, 33, 34] and worsen glycemic control in previously diagnosed diabetes patients [31]. The latter situation may therefore warrant adjustment in doses of antiglycemic agents or a complete switch to insulin therapy.

## 3. The Underlying Pathophysiological Mechanisms

**Figure 1:**
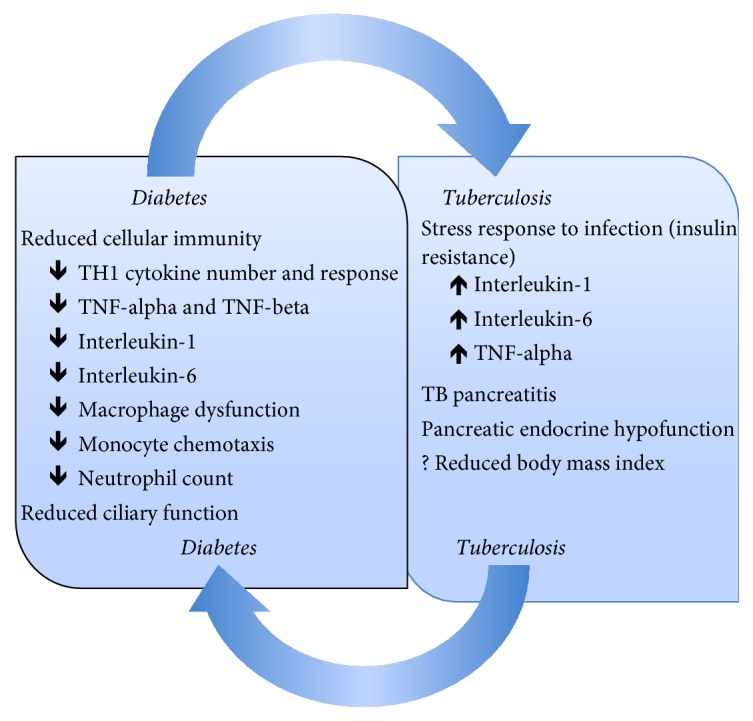
Pathophysiological mechanisms underlying TB–diabetes interaction [3, 18, 28, 29, 31, 35–39]. TH1: T-helper 1; TNF: tumor necrosis factor; TB: tuberculosis.

The increased risk of TB among diabetes patients is multifactorial [35–37] and several putative mechanisms have been proposed (refer to Figure 1). There is decreased cellular immunity due to reduced T-lymphocyte count as well as function and a low neutrophil count [35]. Diabetics show a reduced T-helper 1 (TH 1) cytokine response level, tumor necrosis factor (TNF-alpha and TNF-beta), interleukin-1, and interleukin-6 production compared to their nondiabetic counterparts [35, 36]. The susceptibility of diabetes patients to TB is mainly due to reduced numbers and function of T-lymphocytes. particularly TH1 cytokine inhibition of* Mycobacterium tuberculosis* [35, 36]. There is macrophage dysfunction in diabetes which results in impaired production of reactive oxygen species and phagocytic and chemotactic function [35, 36]. Chemotaxis of monocytes is also impaired in patients with diabetes, a defect which does not improve with insulin [38]. Hyperglycemia is thought to also impair the force of respiratory burst in expelling pathogens [35, 36]. Whilst these proposed mechanisms are plausible, it is important that further mechanistic studies are done to confirm them or otherwise.

The stress response to infection may also play a role in dysglycemia, a situation mediated by the effect of interleukin-1 (IL-1), interleukin-6 (IL-6), and TNF-alpha [3, 31, 39]. This temporal relationship has been demonstrated in some studies where between 19 and 42.6% of active TB patients were discovered to have IGT or diabetes with a significant reduction or complete regression in the rates following treatment [28, 29]. One of the studies had a similar rate of glucose intolerance in the control group who had community-acquired pneumonia, further supporting the possibility of a stress response to infection [29]. In contrast, in the study by Zack et al. [40], 41% of 256 patients admitted to the TB ward had glucose intolerance when oral glucose tolerance tests were performed after at least one month of hospitalization. A larger number of patients continued to have glucose intolerance with some developing diabetes. However, it is believed that the largely abnormal test results, obtained a month after treatment was commenced, may reflect underlying true glucose intolerance rather than a stress reaction to infection [40].

On the other hand, TB may cause TB pancreatitis as well as pancreatic endocrine hypofunction which may lead to IGT or new onset diabetes or worsen its control [31, 39]. TB pancreatitis may become obvious only after the person develops diabetes [31, 39]. Lastly, whilst malnutrition has been proposed as a risk factor for infections and dysglycemia, body mass index has not been associated with IGT or diabetes [18, 28, 29].

## 4. Management of Comorbid Diabetes and TB

Despite suggestions that diabetes may lead to more severe disease, death, and relapse, the dosage regimen and duration of anti-TB drugs among those with or without TB are not different [1, 25]. Traditionally, most centers treat TB for six months, comprising an initial intensive phase of 2 months of rifampicin, isoniazid, pyrazinamide, and ethambutol and a further 4-month continuation phase of rifampicin and isoniazid [1].

There is a proposed pharmacokinetic and pharmacodynamic interaction between anti-TB drugs and antiglycemic agents. Rifampicin, which is key among the cocktail of anti-TB drugs, through enzyme induction, accelerates the metabolism of sulphonylureas and biguanides, reducing their plasma levels and thereby leading to hyperglycemia [3, 41]. Among nondiabetics, it enhances the intestinal absorption of glucose [41]. Isoniazid antagonizes the action of sulphonylureas and worsens glycemic control [34]. In some situations, isoniazid decreases the metabolism of oral antiglycemic agents and increases their plasma levels, such as cytochrome P2C9 (CYP2C9) involved in the metabolism of sulphonylureas; however, it is thought that the inducing effect of rifampicin far outweighs this inhibitory effect [42]. Again, it can inhibit the release of insulin even among nondiabetics causing hyperglycemia [34]. Rifampicin and isoniazid are not known to affect the breakdown of insulin significantly since insulin is mostly degraded by the hydrolysis of disulphide bonds through the action of insulin degrading enzyme in the liver [43].

Theoretically, dipeptidyl peptidase (DPP) IV inhibitors may cause immune paresis and possibly worsen treatment outcomes in TB management [3, 44]. Thiazolidinediones may be substrates for the cytochrome P450 enzymes, which are induced by rifampicin. Rosiglitazone is metabolized largely by CYP2C8, and rifampicin decreases concentrations of rosiglitazone by 54–65% and pioglitazone by 54% [21].

The treatment of diabetes with concomitant TB infection requires careful evaluation and choice of antiglycemic medication. Again, the general approach to management of diabetes does not differ in the presence of TB or not, despite the possible drug–drug interactions described above [1, 3].

Appropriate diet advice is needed, taking into consideration the need to balance glycemic control and the nutritional demands of largely underweight and malnourished individuals [45]. Metformin remains the first-line antiglycemic agent, a relatively safe and cheap drug with reduced incidence of hypoglycemia [45]. Other agents to be considered include sulphonylureas, meglitinides, alpha-glucosidase inhibitors, dipeptidyl peptidase (DPP) IV inhibitors, glucagon-like peptide (GLP) 1 analogs, thiazolidinediones, and insulin [45]. The particular medication choice must be based on patients' characteristics, availability, and cost as well side effects profile. Indeed, treatment must be individualized [45]. Treatment can be escalated in terms of increasing dosages or frequency of a particular class or the addition of one or more classes as the situation may warrant to achieve adequate glycemic control or targets [45].

In many situations, insulin is the preferred agent in type 2 diabetes where there is active TB infection [3]. The rationale for the choice of insulin includes the severe TB infection, body tissue loss, the need for increased anabolism, pancreatic hypofunction, interaction between oral antidiabetic agents and some antituberculous medications as indicated above, and the possibility of associated liver disease which would preclude the use of oral agents [3, 33].

For the reasons above, patients with preexisting diabetes on oral agents may be switched to insulin therapy in active TB once diagnosis is made, or if on insulin already; adjustments might have to be made for worsening glycemic control. Once glucotoxicity improves and infection is controlled, insulin requirement may fall. However, requirement may rise again once appetite improves and food intake increases [3]. The choice of insulin should be based on safety, effectiveness, cost, and patient characteristics. It must be stated, however, that once the infection is controlled, oral antidiabetic agents may be carefully considered [3]. Despite these advantages, insulin may not be readily available or expensive to afford in some parts of the world [46].

For optimum control, regular glucose monitoring is needed. This helps in the early recognition of possible side effects such as hypoglycemia from some antiglycemic medications like sulphonylurea and insulin and the spatial trend of glucose profiles that may need dose adjustments. However, in many parts of the world such as in developing countries, individuals are required to pay for glucometers and glucose strips and there are no compensation schemes or reimbursement. This hampers the ability to achieve targets and identify and confirm hypoglycemia.

Above all, patient education is key in understanding the disease nature (both TB and diabetes), duration of treatment, side effects of drugs, and complications of disease as well as the promotion of healthy lifestyle choices [45, 47, 48].

## 5. Limitations

The impact and relationship between TB and diabetes will vary across different regions of the world depending on the incidence and prevalence of each condition. Diabetes is most prevalent in the western developed world with relatively lower prevalence of TB [2]; it is expected that the incidence of TB among diabetes patients would be low whilst other respiratory infections may be more important [4]. In the developing world with the highest number of both TB cases and projected increase in diabetes, the interaction and the incidence of TB among diabetes would be far higher [2, 4]. Compared to developing countries, resources for the management of these two conditions are also skewed in favor of the western and other more developed countries, a situation which may further compound this expected negative interaction of comorbid diabetes and TB on each other.

## 6. Conclusion

There is a bidirectional relationship between TB and diabetes, and they both impact the presentation of each other [3]. Diabetes is being increasingly recognized as a risk factor for TB and may affect its presentation, whilst TB may worsen glycemic control or lead to IGT among TB patients [3]. This relationship demands adjustment in treatment and the need to employ insulin in the treatment of hyperglycemia during active TB infection when required. A review of the antiglycemic agent(s) is also warranted once TB treatment is over [3].

The expected rise in diabetes cases in developing countries (driven mainly by type 2 diabetes) which also bear the brunt of tuberculosis would increase the influence of diabetes on TB in the coming future [1, 2].

It is hoped that this review would provide further insights into whether routine screening for dysglycemia should be done for all TB patients, especially at the time of diagnosis. Among patients with diabetes, there is a stronger case to be made for screening for TB routinely or at the least suspicion [48]. Diagnosed patients should promptly be referred to a TB center for treatment. Strategies for the prevention of TB must continually be emphasized [48]. These include improvements in housing and nutrition, poverty reduction, treatment of HIV/AIDS, and above all the availability of diagnostic tools such as sputum smear microscopy, X-rays, and automated molecular tests [1, 48].

The realization of these efforts and strategies for the prevention and management of comorbid diabetes will be a challenge in less developed parts of the world [1, 48]. These nations have the least healthcare resources which will hamper their ability to deal with the expected negative impact of diabetes on TB and vice versa [3, 48].

## Abbreviations

AIDS: Acquired immune deficiency syndrome
CI: Confidence interval
CYP: Cytochrome P
DPP IV: Dipeptidyl peptidase IV
HIV: Human immunodeficiency virus
IDF: International Diabetes Federation
IGT: Impaired glucose tolerance
IL: Interleukin
MDR: Multidrug-resistant tuberculosis
OGTT: Oral glucose tolerance test
OR: Odds ratio
ORLS: Oxford Record Linkage Study
RR: Relative risk
TB: Tuberculosis
TNF: Tumor necrosis factor
TH: T-helper
USA: United States of America
WHO: World Health Organization.
